# Catheter Ablation of Atrial Fibrillation in Patients with Heart Failure: Focus on the Latest Clinical Evidence

**DOI:** 10.3390/jcm13175138

**Published:** 2024-08-29

**Authors:** Andrea Demarchi, Matteo Casula, Ginevra Annoni, Marco Foti, Roberto Rordorf

**Affiliations:** 1 Istituto Cardiocentro Ticino, Ente Ospedaliero Cantonale, 6900 Lugano, Canton Ticino, Switzerland; 2 Istituto Cardiocentro Ticino, Ente Ospedaliero Cantonale, 6500 Bellinzona, Canton Ticino, Switzerland; 3 Cardiology and Cardiovascular Intensive Care Unit, ARNAS “G. Brotzu”, 09047 Cagliari, Italy; 4 Department of Molecular Medicine, University of Pavia, 27100 Pavia, Italy; 5 Arrhythmias and Electrophysiology Unit, Division of Cardiology, IRCCS Fondazione Policlinico S. Matteo, 27100 Pavia, Italy

**Keywords:** atrial fibrillation, catheter ablation, heart failure, preserved ejection fraction, reduced ejection fraction

## Abstract

Atrial fibrillation and heart failure are two common cardiovascular conditions that frequently coexist, and it has been widely demonstrated that in patients with chronic heart failure, atrial fibrillation is associated with a significant increase in the risk of all-cause death and all-cause hospitalization. Nevertheless, there is no unanimous consensus in the literature on how to approach this category of patients and which therapeutic strategy (rhythm control or frequency control) is the most favorable in terms of prognosis; moreover, there is still a lack of data comparing the different ablative techniques of atrial fibrillation in terms of efficacy, and many of the current trials do not consider current ablative techniques such as high-power short-duration ablation index protocol for radiofrequency pulmonary vein isolation. Eventually, while several RCTs have widely proved that in patients with heart failure with reduced ejection fraction, ablation of atrial fibrillation is superior to medical therapy alone, there is no consensus regarding those with preserved ejection fraction. For these reasons, in this review, we aim to summarize the main updated evidence guiding clinical decision in this complex scenario, with a special focus on the most recent trials and the latest meta-analyses that examined the role of catheter ablation (CA) in rhythm control in patients with AF and HF.

## 1. Introduction

Atrial fibrillation (AF) and heart failure (HF) are two common cardiovascular conditions that frequently coexist. The pathophysiology of their interrelationship is complex and not fully understood, and they mutually potentiate each other in a bidirectional way. In patients with AF, HF is the most frequent complication (occurring four times more often than stroke) and the most common cause of death [1,2]. On the other hand, about one-third of patients with HF with reduced ejection fraction (HFrEF) also have AF [3,4], while in patients with HF with preserved ejection fraction (HFpEF), the prevalence of AF can reach up to 50% [5]. HFpEF and AF share numerous risk factors (e.g., arterial hypertension, obesity, advanced age and sleep apnea) and have several common pathogenic elements, such as structural alterations (e.g., atrial dilation, myocardial fibrosis and electrical remodeling) and hemodynamic changes (e.g., left ventricular diastolic dysfunction and systolic ventricular–vascular uncoupling). These factors create a vicious cycle where HFpEF and AF perpetuate each other. Additionally, atrial dilation leads to mitral/tricuspid insufficiency, which promotes the onset of HFpEF. Recently, a role for systemic inflammation in the common pathogenesis of these two conditions has also been proposed [6]. Other factors, such as tachycardia-induced cardiomyopathy and neurohormonal activation (upregulation of the renin–angiotensin–aldosterone system and natriuretic peptides), seem to be more involved in the genesis of HFrEF. Moreover, in HF patients, all the three key points of Coumel’s triangle are usually altered [7,8]: the atrial substrate is frequently altered as a consequence of HF itself or due to frequent comorbidities such as arterial hypertension, with the presence of atrial scars, atrial remodeling and upregulation of pro-inflammatory cytokines [9]; studies have also correlated the area of atrial late gadolinium enhancement with rotors localization [10]. Modulating factors and triggers such as sympato-vagal imbalance and LA focal triggers are also more frequent in patients with coexisting HF.

The coexistence of AF and chronic HF in the same patient has negative prognostic implications and makes the treatment of both AF and HF more challenging [11]. 

As pointed out by the results of the COMET [12] trial data, in patients with chronic HF, AF is independently associated with a significantly increased risk of composite all-cause death and all-cause hospitalization. Moreover, new-onset AF was an independent predictor of all-cause death in a time-dependent analysis regardless of treatment allocation and changes in the New York Heart Association (NYHA) class. 

On the other hand, the presence of AF may hamper the prognostic benefit of some HF treatments, such as beta-blockers, or prevent the proper functioning of cardiac resynchronization therapy. Moreover, left ventricular systolic disfunction limits the available antiarrhythmic drugs (AADs) for rhythm control strategy primarily to amiodarone, a drug whose potential mid-to-long-term detrimental effects are well known [13,14]; also, the neurohormonal changes in HF can lead to an accelerated development of AF [15].

Therefore, the coexistence of the two conditions poses a real challenge for the cardiologist [16]. In this review, we aim to summarize the main updated evidence guiding clinical decision in this complex scenario, with a special focus on the most recent trials and the latest meta-analyses that examined the role of catheter ablation (CA) in rhythm control in patients with AF and HF.

## 2. The Prognostic Meaning of Rhythm vs. Rate Control Strategies

Whether a rhythm control strategy confers a prognostic advantage over rate control alone in patients with AF and HF has been a matter of debate. Early evidence from the AF-CHF study showed no benefit of rhythm control obtained with electrical cardioversion and AADs over rate control in symptomatic HFrEF patients [17]. More recent evidence, obtained thanks to the diffusion and improvement of CA for AF, has shown that the method used to maintain sinus rhythm matters (Table 1). A pooled analysis of data from 11 randomized studies (RCTs) enrolling 3598 patients showed that rhythm control strategy reduces hospitalizations and confers a survival and quality-of-life benefit in HFrEF patients when implemented with CA but not with AADs. Accordingly, current guidelines from the European Society of Cardiology, as well as the recently published international joint consensus statement on AF ablation [18], recommend that CA is indicated to reverse LV dysfunction in AF patients when tachycardia-induced cardiomyopathy is highly probable, independent of their symptom status (class of recommendation I, level of evidence B) and be considered in selected AF patients with HF with reduced left ventricular ejection fraction (LVEF) to improve survival and reduce HF hospitalization (class of recommendation IIa, level of evidence B). Distinguishing whether AF is a consequence of HF or its cause may be challenging, and sometimes, a conclusive diagnosis can only be obtained after sinus rhythm restoration. Furthermore, even if AF is not the only cause of HF, it can often contribute to its worsening [19,20,21].

The orientation reported by the guidelines is globally consistent with the results of the study by Zafeiropoulos et al. published in April 2024. They conducted a large meta-analysis investigating the clinical outcomes of rhythm vs. rate control strategies: eighteen RCTs were considered with a total of 17,536 patients and a mean follow-up of 28.5 months. Rate control was pursued with the use of beta-blockers, verapamil, diltiazem or digoxin, while rhythm control was obtained with cardioversion, AF ablation or the use of AADs (classes Ia, Ic and III). Among the patients included, 31.9% had paroxysmal AF, the mean left atrial diameter was 44 ± 7.5 mm, and 8.9% of the patients in the rhythm control arm underwent CA. The rhythm control strategy proved to be superior in reducing CV death, stroke and hospitalization for HF and increased the probability of being in sinus rhythm at the end of the follow-up. Despite the fact that in the studied population, there was no significant difference in all-cause mortality comparing the two strategies, within the arm of the rhythm control strategy, the percentage of catheter ablation use across studies was linearly associated with improved CV mortality and hospitalization for heart failure [22]. These results were also confirmed in the subgroup of patients with HF.

The CASTLE-AF trial, which included both paroxysmal and persistent AF patients with LVEF ≤35% and symptomatic HF, showed that CA almost halved the risk of dying and being hospitalized for worsening HF compared with optimal medical treatment [23]. More recently, a sub-study of the CABANA trial on patients with HF, the majority with preserved LVEF, confirmed, with intention-to-treat analysis, that the use of CA was associated with a reduction of nearly 50% in the risk of death [24]. On the other hand, the RAFT-AF trial, one of the largest studies comparing clinical outcomes between CA and medical therapy in HF patients with AF, failed to show a statistically significant reduction in the risk of all-cause mortality or HF events (29% relative risk reduction with *p* = 0.066) [25]. 

Considering the non-uniform results of the RCTs conducted, Zhang et al. [26], in March 2024, published a large meta-analysis of RCTs with the aim of comparing the long-term outcomes of CA with medical therapy in patients with HF. The main focus was on the impacts on all-cause mortality, the rate of HF hospitalization, stroke, left ventricular function, quality of life and recurrence of atrial tachyarrhythmia (AT). Nine RCTs were included for a total of 2293 patients with HF and AF. In the CA arm, all the patients underwent pulmonary vein isolation, and in the majority of the cases of additional linear and complex fractionated electrograms, ablation was also performed. In the medical therapy arm, both rhythm and rate control strategies were considered. Compared with medical therapy, CA significantly reduced all-cause mortality (RR: 0.60, 95% CI: 0.48–0.74, *p* < 0.01) and HF rehospitalization (RR: 0.65, 95% CI: 0.45–0.94, *p* = 0.02); moreover, there were a greater improvement in left ventricular ejection fraction (MD: 6.26%, 95% CI: 4.18% to 8.34%, *p* < 0.00001) and significantly less recurrence of AT (RR: 0.37, 95% CI: 0.26–0.52, *p* < 0.00001). No significant difference in the rates of stroke and adverse events between CA and medical therapy was observed. These results are globally consistent with those published by our group in 2023 in a meta-analysis that included both randomized and observational studies, reinforcing the message that CA can confer a benefit even outside of the super-selected RCT population [27]. 

Furthermore, Zhang et al. [26] claim that in the RAFT trial, compared with the other eight RCTs considered, there was a stricter control of frequency (mean HR < 80 bpm at rest and <110 bpm during 6 min waking test—6MWT) with medical therapy than in the other trials. Although a post hoc analysis of the RACE II study showed that tight rate control compared with lenient control had no effect on mortality and hospitalization in patients with HFpEF [28]; a lower heart rate at baseline and during exercise may have attenuated the benefits of CA in the trial. 

The mechanism by which sinus rhythm confers a prognostic benefit in patients with HF, therefore, probably goes beyond a simple reduction in the heart rate. At least two other main determinants should be considered: irregular heart rate and loss of atrial systole. The role of the first mechanism, which leads to altered calcium dynamics [29], is highlighted by the results of the APAF-CRT trial, revealing that patients treated with atrioventricular nodal ablation plus biventricular pacing had lower risk of all-cause mortality compared with pharmacological rate control (HR 0.26, 95% CI 0.10–0.65, *p* = 0.004) [30]. Loss of atrial contractility and emptying may trigger sympathetic activation, leading to restricted ventricular filling and diastolic dysfunction with increased left ventricular filling pressures. Moreover, energy deficit and metabolic dysregulation with mitochondrial dysfunction have been reported in the atrial myocardium of patients with HF, which is exacerbated in the presence of AF [31]. 

The additional benefit conferred by AF ablation compared with atrioventricular node ablation and biventricular stimulation was evaluated more than 15 years ago by the PABA-CHF trial. Patients with symptomatic and drug-resistant AF, an ejection fraction of 40% or less and NYHA class II or III were randomized to undergo either pulmonary vein isolation or atrioventricular node ablation with biventricular pacing. The pulmonary vein ablation strategy was shown to be superior to atrioventricular node ablation with biventricular pacing considering the composite primary endpoint of improvement in ejection fraction, distance on the 6 min walk test and Minnesota Living With Heart Failure (MLWHF) score [32]. The latter consists of 21 questions regarding how HF is impacting the patient’s life, with a higher score indicating a worse quality of life. In the study, in the group that underwent PVI, the mean score improved significantly compared with AV node ablation and BIV pacing. However, no data on hard endpoints were evaluated, and nowadays, new studies evaluating the comparison between pulmonary vein isolation using the latest ablative technologies and atrioventricular node ablation with conduction system stimulation would be necessary to clarify this point. 

Although the role of CA in patients with HF and AF is increasingly consolidated, the importance of optimized management of HF and guideline-directed medical therapy should never be underestimated, also to optimize rhythm control, as also shown by the results of the RACE 3 trial [33]. 

## 3. The Effect of CA on LVEF

Another important issue that has been debated in recent years is the role of CA in improving LVEF after CA.

The CAMERA MRI study was designed to specifically address this question in patients in whom the etiology of the underlying LV dysfunction was otherwise unexplained apart from the presence of AF. In the primary analysis, the authors showed an absolute LVEF improvement of 18 ± 13% in the catheter ablation (CA) group compared with 4.4 ± 13% in the medical rate control arm (*p* < 0.0001). Normalized LVEF (defined as >50% after treatment) was observed in 58% of the cases after CA vs. 9% in the control group (*p* = 0.0002). They also sought to further assess the subgroup of patients who were more likely to experience LVEF recovery after CA, and they found that the absence of ventricular fibrosis (identified as late gadolinium enhancement upon nuclear magnetic resonance scan) was a predictor of greater LVEF improvement after CA [34].

Interestingly, the CAMERA-MRI Long Term Outcome published after a 4 ± 0.9 years follow-up confirmed their previous results, with a long-term absolute increase in LVEF of 16.4 ± 13.3% in the CA group [35]. 

In contrast, the AMICA trial did not prove any benefit of CA in patients with AF and advanced HF, mainly because at 1 year, LVEF increased in a similar fashion in patients treated with CA and patients treated with optimal medical therapy alone (8.8% vs. 7.3%). Moreover, they showed no statistical differences in the secondary endpoint of the quality-of-life score, nor NT-pro-BNP or 6MWT changes between patients in the CA arm vs. medical arm [36]. 

A possible explanation for the contradictory results of these studies may reside in the heterogeneous population of HF patients enrolled and in the different underlying structural heart disease in different subgroups. To overcome these limitations, the ANTWERP SCORE has been developed to help stratify HF patients who will likely benefit from CA. This prediction model, based on four predictors, i.e., QRS width, known etiology of HF, AF pattern and atrial dimension, was validated both in a single-center cohort, as well as a large multicenter cohort, and effectively predicted LVEF recovery after CA and discriminated clinical outcomes [37,38].

## 4. HF and CA Technique

Data regarding ablative technique comparisons in patients with AF and HF are scanty. Although the two landmark randomized controlled trials comparing cryoablation (CB) vs. radiofrequency pulmonary vein isolation (RF PVI) (i.e., FIRE and ICE trial and CIRCA-DOSE) showed the non-inferiority of CB in achieving rhythm control, they both enrolled patients with paroxysmal AF and mostly normal LVEF and also omitted some current technology, such as high-power short-duration ablation index protocol for RF PVI [39,40]. These findings are also observed in the large meta-analysis published by our group comparing CB and RF PVI as the first ablation procedure for AF [41]. Any extrapolation for the current HF population and any direct comparison between the contact force and ablation index-guided RF PVI and second-generation CB PVI in this subgroup of patients is, therefore, impossible.

Moreover, AF ablation in patients with reduced LVEF may also be technically more challenging due to the presence of augmented filling pressure, the frequent presence of enlarged left atrium, atrial scar and larger pulmonary vein ostia. Due to these anatomical and pathophysiological features, some considerations, although speculative, can be made: Even if no randomized data show net clinical benefit in performing RF substrate atrial ablation beyond PVI in an unselected population of persistent AF patients, it may be speculated that RF, giving the possibility of more extensive atrial substrate ablation in HF patients, could lead to better results than CB. Meanwhile, CB could lead to larger posterior wall area isolation close to the PV ostia and may also lead to a larger effect on autonomic ganglia modulation [42], whose role in maintaining AF in HF patients has been demonstrated.

As stated above, direct comparisons of procedure techniques is lacking.

Considering the potential greater technical challenge posed by PVI in patients with reduced LVEF for the reason stated above, the question could be raised whether CA in this population is associated with lower success in preventing AF recurrences.

Demarchi et al. sought to assess the efficacy of CA in patients with reduced LVEF and severe left atrial enlargement, and they compared it to that in patients with preserved left ventricular function and equally severe dilated left atrium. Their results show that success rates in maintaining sinus rhythm observed after a first procedure were not inferior to those for patients with normal systolic function, thus confirming that a low LVEF does not preclude a successful outcome [43].

Similar results were found by Rordorf et al. [44] in a much larger cohort. The Cryo AF Global registry showed that freedom from repeated procedure was not different between patients with HF vs. no-HF; moreover, a reduction in AF-related symptoms and antiarrhythmic drugs use was also non-statistically different between patients with and without HF after ablation [44].

These results were confirmed in a recently published meta-analysis. Besides several limitations, such as the lack of stratification for HF and AF types and the absence of randomized data, in the 307 patients (HFrEF) analyzed from the five observational studies included, Tokavanich and colleagues showed an AF freedom rate at 1 year of 64%, comparable or even superior to data regarding RF success rates in patients with AF from the literature. Moreover, the risk of recurrence of atrial arrhythmia was not significantly different between HF and no-HF patients (RR 1.34, 95% CI 0.8–2.23) [45].

Very few data are available regarding pulsed field ablation (PFA) in HF [46] because usually patients with reduced EF were only a tiny fraction of patients involved in the first studies with this technique and no specific trail enrolling patients with HFpEF has been made yet. In a sub-study of the MANIFEST-PF, a multicenter patient-level registry of consecutive patients undergoing PFA for paroxysmal or persistent AF, patients were stratified as no-HF, HF with preserved EF (LVEF ≥ 50%) or HF with reduced/mildly reduced EF (LVEF < 50%). Out of the 1381 patients enrolled, only 6.2% matched criteria for HFpEF and 8.6% for HFrEF or HFmrEF.

The rate of 1-year freedom from atrial arrhythmia was significantly higher in no-HF patients than in HFpEF or HFrEF and HFmrEF patients (79.9%, 71.3% and 67.5%; *p* < 0.001) but similar between HFmrEF and HFpEF patients [47].

Current data on this issue are still too few and partial. Understanding whether PFA is truly associated with worse performance in patients with HF will require further specific investigations.

## 5. CA across the LVEF Spectrum: HFpEF

While evidence from new trials seems to be moving toward a trend favoring CA over drug therapy in patients with HF in general, it should not be forgotten that within the nosology of HF, we recognize three categories according to the value of LVEF: HF with reduced ejection fraction (HFrEF—LVEF ≤ 40%), mildly reduced ejection fraction (HFmrEF—LVEF 41–49%) and preserved ejection fraction (HFpEF—LVEF ≥ 50%). Patients belonging to these categories can be very different from each other, in terms of etiology, pathophysiology and course of the disease. In the wake of the limited effectiveness of HFrEF drug therapy in HFpEF patients, it is legitimate to wonder whether the trend to move toward a more aggressive approach with CA as first-line therapy in AF is effective for all categories of patients with HF or whether we should differentiate our approach according to patient phenotype, as we already do for drug therapy. 

For patients with HFrEF, it is now established from RCTs, systematic reviews and meta-analyses that CA of AF reduces mortality and hospitalization and improves prognosis compared with medical therapy [48]. On the other hand, despite HFpEF being destined to become the predominant form among patients with HF and also being the one in which the incidence of AF is the highest, the evidence on the prognostic benefit of AF ablation in patients with HFpEF is weaker (Table 2). No specific randomized study has ever been conducted in the HFpEF population regarding hard endpoints, and the available evidence comes from observational studies, post hoc analyses of RCTs and their meta-analyses. The only randomized evidence comes from a study by Chieng et al. in a total of 31 patients with the aim to assess the effect of AF ablation vs. medical therapy on markers of HF severity, natriuretic peptide level and perceived HF symptoms [49]. Patients underwent exercise right heart catheterization (ExRHC), with a ballon-tipped catheter placed in the pulmonary artery via right brachial or jugular vein, and cardiopulmonary exercise testing (CPET). The primary endpoint of the study was the difference in peak post-capillary wedge pressure (PCWP) on ExRHC from baseline to 6 months. Secondary outcomes included differences in CPET parameters, differences in QoL measurements and differences in natriuretic peptide values from baseline to 6 months. Compared with optimal medical therapy, CA was associated with PCWP improvement, reduction in natriuretic peptide levels and improvements in AF and HF symptoms and quality-of-life questionnaire scores. Moreover, 50% of patients in the ablation arm presented a reversal of HFpEF criteria with normalization of PCWP.

The post hoc analysis of the CABANA trial is the largest dataset available to date comparing, in a controlled setting, CA versus drug therapy in patients with HF and AF. Baseline LVEF values were available for 571 out of 2204 patients randomized in the trial. Of these, 79% had an LVEF ≥50%, 11.7% had an LVEF between 40% and 49%, and 9.3% had an LVEF <40%. In the post hoc analysis, after employing multiple imputation to impute missing baseline LVEF values, CA reduced mortality by 60% relative to drug therapy in the patients with LVEF ≥50% (HR 0.40, 95% CI 0.18 to 0.88) with 4-year Kaplan–Meier mortality rates of 3.3% vs. 8.6%. 

Bulhoes et al., in 2024, conducted a systematic review of eight studies (three post hoc analysis or subgroup analyses derived from RCTs and five observational studies) with a total of 20,257 patients with a follow-up ranging from 24.6 to 61.2 months. The outcomes were a composite of death and hospitalization for heart failure, all-cause death, cardiovascular death and all-cause rehospitalization. Patients treated with ablation had a significantly lower risk of all-cause death, cardiovascular death and hospitalization, both for decompensated HF and for all causes. The hemodynamic changes related to the effective maintenance of sinus rhythm (reduction in atrial dilatation and fibrosis with development of mitro-tricuspid insufficiency, reduction in increased filling pressures and improvement in both systolic and diastolic function) were so significant that about 50% of patients no longer met the hemodynamic criteria for HFpEF [56].These results are in contrast to the systematic review published by Oraii et al. in April 2024, where 12 RCTs were considered with a total of 2465 patients with both HFrEF and HFpEF, comparing the efficacy of transcatheter ablation with medical therapy. While the study reported a marked reduction in the risk of cardiovascular events and cardiovascular death in patients with HFrEF, the same results were not observed in patients with HFpEF [57]. However, the *p*-values for the interaction were not significant, thus preventing us from drawing conclusions about a significantly different effect of ablation compared with medical therapy in the two subgroups studied (i.e., HFrEF vs. HFpEF). The body of evidence is globally in agreement, suggesting a prognostic benefit of ablation also in patients with HFpEF. 

Further RCTs specifically designed to evaluate the benefit on hard endpoints of CA compared with medical therapy in patients with HFpEF will be necessary before considering ablation as first-line therapy regardless of symptoms in this patient population.

## 6. CA across the LVEF Spectrum: End-Stage HF

Catheter ablation appears to play a significant role even in patients with end-stage HF. The CASTLE-HTx [58], a single-center, open-label, investigator-initiated, superiority, randomized clinical trial, enrolled a total of 194 patients with advanced heart failure (NYHA class II or higher, LVEF of 35% or less and impaired functional capacity as assessed by the 6MWT) and symptomatic AF, referred for heart transplantation evaluation, who were assigned in a 1:1 ratio to receive either CA or medical therapy alone. Both groups received optimized medical therapy according to guidelines. The primary endpoint was a composite of death from any cause, implantation of a left ventricular assist device or urgent heart transplantation. Investigators planned for a 3-year follow-up, but after 1 year, the data safety monitoring board recommended termination of the trial due to substantial benefits observed in the ablation arm. After a median follow-up of 18 months, a primary endpoint occurred in 8% of patients in the ablation arm versus 30% in the medical therapy arm (hazard ratio, 0.24; 95% CI, 0.11–0.52; *p* < 0.001). Death from any cause occurred in 6% of the ablation group versus 20% of the medical therapy arm (HR, 0.29; 95% CI, 0.12–0.72). Additional endpoints favored ablation over medical therapy, including improvement in left ventricular function, reduced AF burden and less use of amiodarone. Procedure-related complications were minimal and included only groin access issues.

These results, showing a significant improvement in hard outcomes with CA, are particularly important, considering that the cohort of patients with end-stage HF eligible for heart transplantation were excluded from major trials, leaving them with no recommendations or evidence for the optimal treatment of AF. Moreover, the trial enrolled a cumulative 69% of patients with NYHA III-IV, with 14% of patients in the NYHA IV stage, usually excluded in other trials. Noteworthy is how this result was obtained without the complete elimination of AF episodes but only guaranteeing AF burden reduction from 50.9 ± 31.2% to 19.6 ± 28.0% 12 months after CA. 

However, some limitations of this study should be considered: The early termination of the study does not allow us to assess the long-term follow-up of these patients, which could differ from the results at 18 months. The open-label design might also have influenced treatment decisions regarding the components of the primary endpoint. Sixteen patients in the medical-therapy group crossed over to undergo catheter ablation. Out of 900 patients evaluated, only 194 were enrolled, excluding a large portion of patients with advanced HF. Despite the doubts these considerations may raise about the possibility of extending the study results to larger samples, it is evident that AF can clinically deteriorate patients with advanced HF and that CA might represent a valid therapeutic weapon. Undoubtedly, further trials are needed to explore this topic and define the best therapeutic strategy.

## 7. Healthcare Cost Implications

The strict relationship between AF and HF has been also proven to have a significant impact on healthcare costs. The study published by Field et al. was the first real-world study assessing the relationship between CA and healthcare resource utilization in patients with both AF and HF. Based on a large United States administrative database, a significant reduction in AF-related resource use up to three years after CA was clearly demonstrated; moreover, cost reduction was not affected by the need of redo procedures. This may be explained in the significant cost reduction for fewer repeat cardioversion procedures, fewer emergency department access instances for worsening HF and fewer ambulatory care visits in the subgroup undergoing CA [59].

## 8. Conclusions

The coexistence of AF and HF in the same patient is mediated by complex and incompletely understood pathophysiologic mechanisms, leading to negative prognostic implications and making the treatment of both AF and HF more challenging for the cardiologist. In recent years, several observational and randomized studies have evaluated what the best therapeutic strategy might be for this patient population. It is becoming increasingly evident how rhythm control strategies can confer a prognostic advantage over rate control alone in patients with AF and HF, especially when achieved by using CA. In patients with HFrEF, the ablative approach has indeed proven to be globally superior to medical therapy in reducing mortality (all-cause and cardiovascular), hospitalizations for HF, as well as leading to an improvement in symptoms, LVEF, functional capacity and maintenance of sinus rhythm. Although available evidences suggest a similar benefit in terms of hard endpoints in patients with HFpEF, these evidences mainly come from observational studies, post hoc analyses of RCTs and their meta-analyses. Further randomized clinical trials, specifically designed to test CA for each HF phenotype, are then required.

## Figures and Tables

**Table 1:** Atrial fibrillation ablation in HFrEF.

	CASTLE-AF	RAFT-AF	CASTLE-HTX	AMICA TRIAL	CAMERA- MRI
Year	2018	2022	2023	2019	2017
Design	Multicenter open label RCT	Multicenter open-label RCT	Single-center open-label RCT	Multicenter open-label RCT	Multicenter RCT
Mean age (years)	64 ± 5	67 ± 8	64 ± 11	65 ± 8	61 ± 10
AF type	Parox: 32.5% Pers: 38.3% LS-pers: 29.2%	Parox: 7.3% Pers: 69.3% LS-pers: 23.4%	Parox: 30% Pers: 56% LS-pers: 14%	Pers: 76.4% LS-pers: 23.6%	Pers: 100%
Baseline LVEF	25–38%	41 ± 15%	27 ± 6%	26 ± 9%	33 ± 9%
NYHA	I: 11% II: 60% III: 28% IV: 1%	II: 67% III: 33%	II: 31% III: 55% IV: 14%	II: 39% III: 61%	Mean NYHA class: 2.5 ± 0.6
Adjunctive ablation targets other than PVI	ND	91.2%	ND	33%	100%
Control arm therapy	Medical therapy	Rate control	Guideline directed	Best medical therapy	Rate control
Primary outcome	All-cause mortality and HF hospitalization	All-cause mortality and HF events	Death from any cause, LVAD implantation or urgent heart TX	Absolute increase in LVEF	Change in LVEF at 6 months
Mean change in LVEF (ablation vs. control)	8.0% vs. 0.2%	10.1 ± 1.2 vs. 3.8 ± 1.2	7.8 ± 7.6 vs. 1.4 ± 7.2	8.8% vs. 7.3%	18.3% vs. 4.4%
Rhythm control outcome (ablation vs. control)	63.1 vs. 21.7% in SR (5 years)	85.6 vs. 12.9% in SR at 2 years	31.4 ± 33.3 vs. 8.6 ± 26.3 AF burden reduction at 1 year	73.5% vs. 50% in SR	25% vs. 100% in AF
Main findings	Reduction in all-cause death or HF hospitalization	Similar primary outcomes and increase in LVEF	Reduction in primary composite endpoint	No LVEF improvement	LVEF improvement
Follow-up (months)	38	37	18	12	6

RCT: randomized controlled trial; AF: atrial fibrillation; Parox: paroxysmal AF; Pers: persistent AF; LS-Pers: long-standing AF; LVEF: left ventricular ejection fraction; SR: sinus rhythm; HF: heart failure; LVAD: left ventricular assist device; heart TX: heart transplantation; PVI: pulmonary vein isolation; NYHA: New York Heart Association score.

**Table 2:** Atrial fibrillation ablation in HFpEF.

	CABANA Subanalysis [50]	EAST-AFNET 4 [51]	Xie et al. [52]	Tsuda et al. [53]	Ratkka et al. [54]	Olshausen et al. [55]
Year	2021	2021	2023	2023	2021	2022
Design	RCT post hoc analysis	RCT post hoc analysis	Retrospective, observational	Retrospective, observational	Retrospective, observational	Retrospective, observational
Mean age (years)	68 ± 8	>75	63–76	68.4	61 ± 10	Ablation arm mean age: 67 Non-ablation arm mean age: 77
AF type	Parox: 31.6% Pers: 55.3% LS-pers: 13.1%	Ablation arm: Parox: 33.2% Pers: 32.2% Non-ablation: Parox: - Pers: 40%	Ablation arm: Pers: 63.5% Non-ablation: Pers: 61.8%	Ablation arm: Pers: 77.4% Non-ablation: Pers: 77.4%	Ablation arm: Parox: 60% Pers: 40% Non-ablation: Parox: 51% Pers: 49%	Ablation arm: Parox: 17.1% Pers: 32.7% Non-ablation: Parox: 17.5% Pers: 35.9%
Ablation/no ablation	295/315	224/218	293/293	106/106	43/43	434/868
Control arm therapy	Medical therapy (rate or rhythm control)	Rate control	Medical therapy (rate or rhythm control)	Medical therapy (rate or rhythm control)	Medical therapy (rate or rhythm control)	Medical therapy (rate or rhythm control)
Primary outcome	All-cause mortality, disabling stroke, serious bleeding and cardiac arrest	All-cause mortality and HF events	Death from any cause or HF rehospitalization	Reduction in death or heart failure	Time to death or HF hospitalization	All-cause mortality and first HF hospitalization
Main findings	Reduction in primary composite, all-cause mortality and improvement QoL	Sinus rhythm at 12 months explains 81% of effect of early rhythm control on preventing cardiovascular outcomes	Reduction in primary composite endpoint	Reduction in primary composite endpoint	Reduction in HF hospitalization and HF symptoms and improvement in diastolic function	Reduction in primary composite endpoint
Follow-up (months)	60	37.4	39	24.6	35 ± 22	6.1

RCT: randomized controlled trial; HF: heart failure; QoL: quality of life; Parox: paroxysmal AF; Pers: persistent AF; LS-Pers: long-standing AF.

## Data Availability

No new data were created or analyzed in this study.
